# UCST Type Phase Boundary and Accelerated Crystallization in PTT/PET Blends

**DOI:** 10.3390/polym12112730

**Published:** 2020-11-17

**Authors:** Kousuke Sugeno, Satoshi Kokubun, Hiromu Saito

**Affiliations:** Department of Organic and Polymer Materials Chemistry, Tokyo University of Agriculture and Technology, Koganei-shi, Tokyo 184-8588, Japan; s206182z@st.go.tuat.ac.jp (K.S.); satoshi.kokubun@toyoda-gosei.co.jp (S.K.)

**Keywords:** PTT, PET, blend, crystallization, phase separation, phase diagram

## Abstract

We investigated the structure development and crystallization kinetics in the blends of poly(trimethylene terephthalate) (PTT) and poly(ethylene terephthalate) (PET) by polarized optical microscopy and light scattering. The crystallization of the blend was found to be faster and the size of the spherulites was much smaller than those of the neat component polymers by melt crystallization at low temperature of 180 °C. The discontinuous gap of the crystallization time with temperature was seen in the blends, suggesting phase transition at the temperature *T*_tr_; e.g., the *T*_tr_ of the 60/40 PTT/PET was 215 °C. The crystallization was accelerated due to enhancement of the nucleation rate, and interconnected tiny spherulites were obtained at the temperature below the *T*_tr_. The accelerated crystallization and the development of the interconnected structure might be attributed to the liquid-liquid phase separation via spinodal decomposition, due to existence of the upper critical solution temperature (UCST) type phase boundary.

## 1. Introduction

**Figure 1:**
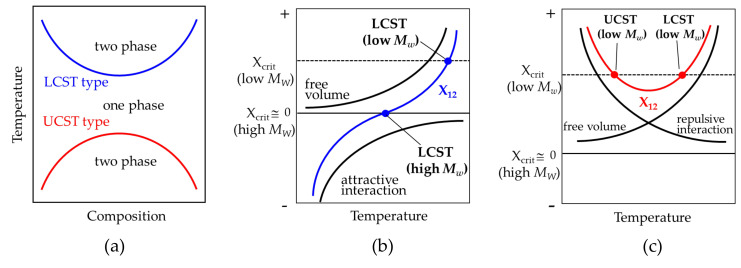
Schematic illustration of phase diagrams and temperature dependence of χ_12._: (**a**) LCST and UCST phase diagrams, (**b**) χ_12_ for LCST type phase behavior and (**c**) χ_12_ for UCST type phase behavior.

Polymer blend is a mixture of two or more dissimilar polymers to create a new material. Most pairs of dissimilar polymers with high molecular weights are immiscible and dissimilar polymers are only miscible when there is favorable specific interaction between them. Miscible polymer blends tend to phase separate at elevated temperatures. This lower critical solution temperature (LCST) type phase behavior (Figure 1a) is typical in miscible polymer blends [1,2,3,4]. Such phase separation occurs when Flory-Huggins interaction parameter χ_12_ becomes larger than the χ_12_ at critical point χ_crit_, by decreasing the negative contribution of the attractive interaction and increasing the positive contribution of the free volume effect arising from the different thermal expansion coefficients of the two components with increasing temperature (Figure 1b). On the other hand, some miscible polymer blends exhibit upper critical solution temperature (UCST) type phase behavior, in which miscible blends tend to phase separate at lower temperature (Figure 1a). Since the χ_crit_ is smaller with increasing molecular weight (*M*w) and is negligibly small in a blend of polymers with high molecular weight, UCST type phase behavior occurs only when the χ_crit_ is large in low *M*w and the positive contribution of the repulsive interaction decreases with increasing temperature (Figure 1c). Hence, UCST phase behavior is expected to be uncommon for a blend of polymers with high molecular weights, and it is observed in oligomer blends and random copolymer blends [3,4,5,6,7,8,9]. UCST type phase behavior is also suggested in crystalline polymer blends [10,11,12,13,14,15,16,17,18,19].

In miscible polymer blends, crystallization rate of the crystalline polymer is drastically delayed and spherulites thus obtained becomes larger and coarser with increasing the content of partner polymer by crystallization from the homogeneous melt [20,21,22,23,24,25,26,27,28,29]. It is considered that the delay of the crystallization rate is attributed to the exclusion of partner polymer from the crystallization growth front [24,28]. When UCST type phase diagram exists below the melting temperature and the blend is crystallized at the temperature below the UCST phase boundary, the crystallization can be accelerated and interconnected crystalline structure is developed due to the cooperative progress of the crystallization and liquid-liquid phase separation via spinodal decomposition, as reported in PVDF/PMMA [11,17] and polycarbonate/PEO blends [15]. Accelerated crystallization and connected crystalline structure are also observed in polyolefin blend such as poly(ethylene-co-hexene) (PEH)/poly(ethylene-co-butene) (PEB) one in which UCST type phase boundary exists above the melting temperature [18,19], and the accelerated crystallization is suggested to be caused by the crystal nucleation enhanced at the diffuse interface between two immiscible polymers [30,31,32].

Poly(trimethylene terephthalate) (PTT) is a crystalline aromatic polyester which exhibits large spherulite with high birefringence [33,34]. Due to its high elastic recovery, PTT is used for fiber in carpet and textile industry, for instance. However, PTT is difficult to use for film application because large shrinkage occurs after elongation in film processing and it is brittle due to large spherulite obtained during melt-cooling process owing to the slow crystallization rate. To prevent the shrinkage and control the crystallization rate, polymer blend method is promising for wide application of PTT. PTT is known to be miscible with crystalline polyester of poly(ethylene terephthalate) (PET) in melt state at the temperature above the melting temperature of PET at entire blend composition [35,36,37,38,39]. Recently, we found that the crystallization of PTT was accelerated at lower temperature by blending PET. In this study, to understand the accelerated crystallization of the PTT/PET blends, we investigated the crystallization kinetics and structure development by light scattering method and optical microscopic observation. The results are discussed in terms of the liquid-liquid phase separation by existence of the phase boundary.

## 2. Materials and Methods 

PTT (intrinsic viscosity IV = 1.7 dl/g) was supplied by Asahi Chemical, Inc. (Tokyo, Japan) and PET (IV = 0.45 dl/g) was supplied by Teijin, Inc. (Tokyo, Japan). Melting temperatures of the PET and PTT were 258 °C and 228 °C, respectively. The PET and PTT were dried in vacuum oven at 100 °C for 1 day before melt mixing, to prevent transesterification reaction during the mixing. The PTT and PET were melt-mixed at a temperature of 300 °C and at rotor speed of 200 rpm for 5 min in a mixing chamber of a miniature mixing machine (Imoto IMC-18D7, Kyoto, Japan). The blend specimens thus obtained were compression-molded between two metal plates at 300 °C for 1 min to obtain a film specimen with a thickness of about 100 μm, which was then quenched into ice water. 

The film specimen thus prepared was melted at 300 °C for 1 min in a hot stage, then was rapidly transferred into another hot stage set on the light scattering stage and annealed at Ta. A polarized He–Ne laser with a wavelength of 632.8 nm was applied vertically to the film specimen. The scattered light was passed through the analyzer and then onto a highly sensitive charge-coupled device (CCD) camera with 800 × 600 pixels (Tokyo Instruments pco.1600, Tokyo, Japan). We employed Hv and Vv geometries in which the optical axis of the analyzer was vertical to that of the polarizer and was horizontal to that of the polarizer, respectively. The input data from the CCD camera were stored in a personal computer for further analysis. 

Crystalline morphologies and phase structure of the specimens were observed both under an unpolarized optical microscope and a polarized optical microscope (Olympus BX53, Tokyo, Japan), each equipped with a CCD camera (Olympus DP73, Tokyo, Japan). The structure under the polarized optical microscope was observed by the optical microscope equipped with a sensitive tint plate, with an optical path difference of 530 nm under crossed polarizers. The development of the crystalline morphologies and phase structure during the annealing was observed under optical microscope equipped with a hot stage set at annealed temperature Ta after rapid transfer from another hot stage set at 300 °C. The melting behavior during the heating was also observed under polarized optical microscope equipped with a hot stage.

## 3. Results and Discussion

**Figure 2:**
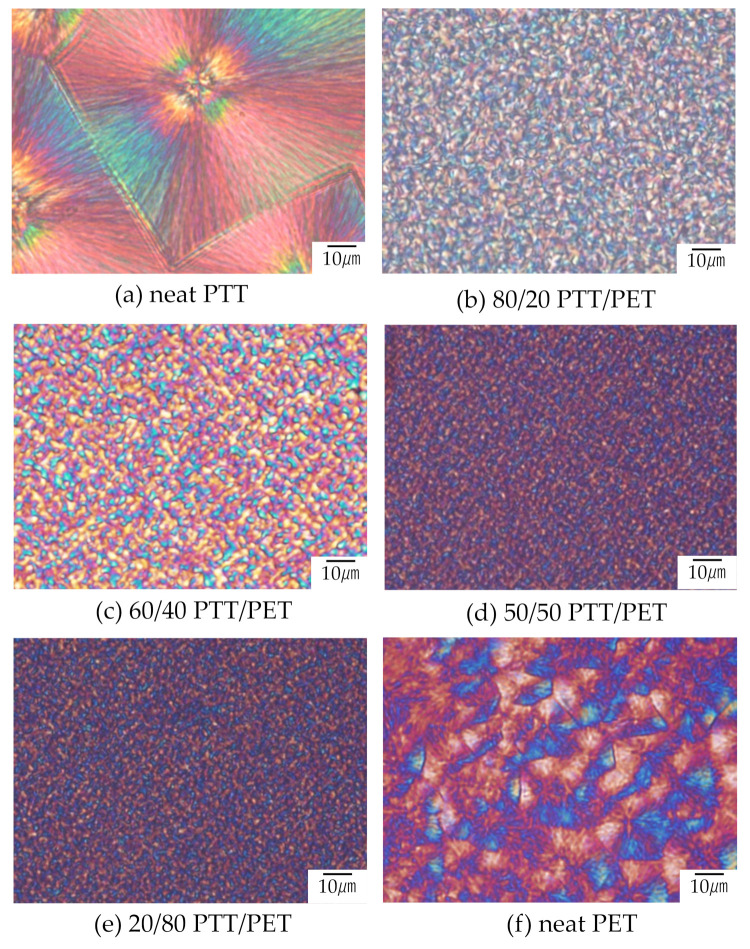
Polarized optical micrographs of (**a**) neat PTT, (**b**) 80/20 PTT/PET, (**c**) 60/40 PTT/PET, (**d**) 50/50 PTT/PET, (**e**) 20/80 PTT/PET and (**f**) neat PET obtained by isothermal annealing at 180 °C from the melt state at 300 °C.

No structure appeared by annealing at the temperature above the melting temperature of poly(ethylene terephthalate) (PET), suggesting that poly(trimethylene terephthalate) (PTT) and PET are miscible and liquid-liquid phase separation does not occur at the temperature above the melting temperatures of PET, as reported in the references [35,36,37,38,39]. By temperature-drop from the homogeneous melt state, crystallization occurred by isothermal annealing at the temperature below the melting temperature of PET. Figure 2 shows the polarized optical micrographs of neat PTT, neat PET and PTT/PET blends obtained by melt crystallization at 180 °C. Large spherulites were obtained in the neat PTT and neat PET (Figure 2a,f). On the other hand, tiny, distorted crystals were formed and were connected each other in the blends (Figure 2b–e). The size of the spherulite was much smaller than those of the neat component polymers, suggesting nucleation agent effect by blending. This result is different from that usually observed in the crystalline morphology of miscible polymer blends in which the spherulite of the blend is much larger than those of the neat component polymers [20,21,22,23,24,25,26,27,28,29]. 

**Figure 3:**
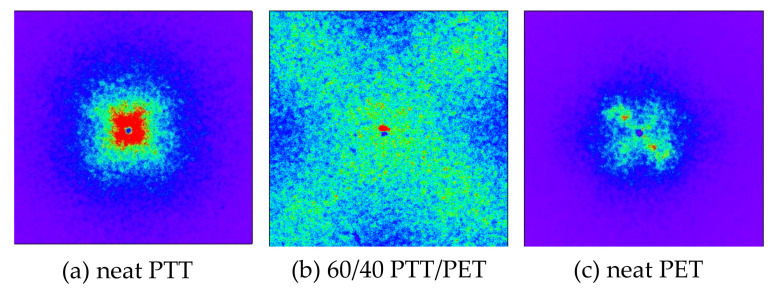
Hv light scattering patterns of (**a**) neat PTT, (**b**) 60/40 PTT/PET and (**c**) neat PET obtained by isothermal annealing at 180 °C.

Figure 3 shows the Hv light scattering patterns of 60/40 PTT/PET and the neat component polymers obtained by melt crystallization at 180 °C, in which the crystalline morphologies are shown in Figure 2. Here the pattern was shown at same scattering angle region for comparison. Four-leaf clover type pattern was seen in the neat PET and the blend, which is characteristic of spherulite with radial arrangement of the crystallites [40], while the pattern of the neat PTT was too small to see at the same scattering angle region. Thus, the clover pattern of the blend was much larger than those of the neat component polymers. Since the size of the clover pattern is larger as the size of the spherulite is smaller [40], the results support the polarized optical microscopic observation shown in Figure 2 that the size of the spherulites of the blend was much smaller than those of the neat component polymers. 

The Hv scattering is attributed to the optical anisotropy of the crystallites. The crystallization kinetics can be discussed by the integrated scattering intensity, i.e., the invariant *Q* defined by

(1) $$\;Q=\int_{0}^{\infty }I\left(q\right)q^{2}dq\;$$

where *q* is the scattering vector, $q=\left(4\pi /\lambda \right)\sin\left(\theta /2\right)$, λ and *θ* being the wavelength and scattering angle, respectively, and *I*(*q*) is the intensity of the scattered light at *q*. As shown in Figure 3, the Hv scattering pattern from the crystallized specimen was a four-leaf clover type, suggesting the scattering from spherulites. In this case, the invariant in Hv mode *Q*_Hv_ is described by the mean square optical anisotropy <*δ*^2^>

(2) $$\;Q_{Hv}\propto {\langle \delta \rangle }^{2}=\phi_{s}\;{\left(\alpha_{r}-\alpha_{t}\right)}^{2}\;$$

where *ϕ*_s_ is the volume fraction of spherulite and α_r_ and α_t_ are the radial and tangential polarizabilities of the spherulite, respectively [15,41,42]. 

**Figure 4:**
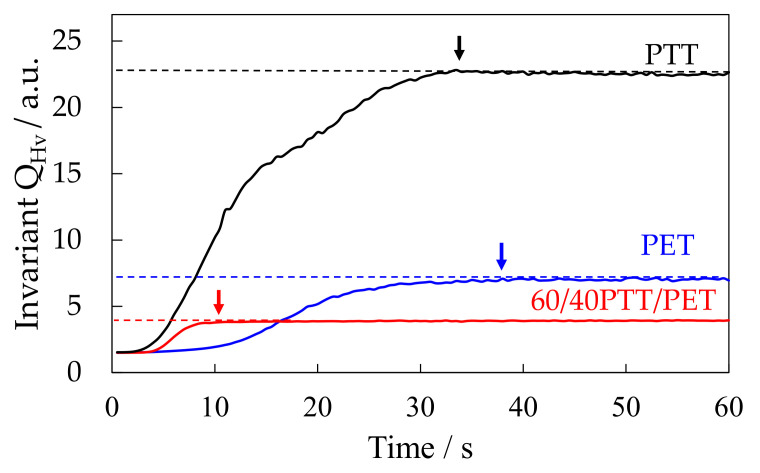
Time variation of invariant *Q*_Hv_ for 60/40 PTT/PET and neat component polymers during isothermal annealing at 180 °C.

Figure 4 shows the time variation of the invariant in Hv mode *Q*_Hv_ in the 60/40 PTT/PET and neat component polymers during annealing at 180 °C after temperature drop from 300 °C. The *Q*_Hv_ increased with time and leveled off as expected from Equation (2), i.e., *ϕ*_s_ increases and attains a maximum when spherulites fill the whole space and crystallization completes. The *Q*_Hv_ of the neat PTT was large due to large polarizability difference α_r_−α_t_ owing to high birefringence in the spherulite. The crystallization of the blend started to occur at around 2 s and completed at 10 s. On the other hands, crystallization of the neat PTT and neat PET started to occur at 1 s and 4 s, respectively, and completed at 34 s and 38 s, respectively. These results indicate that the crystallization rate of the blend is much faster than those of the component polymers. Thus, crystallization of PTT and PET were accelerated at the temperature of 180 °C by blending. It is opposite to the delay of crystallization generally observed in miscible polymer blends [20,21,22,23,24,25,26,27,28,29]. The accelerated crystallization and the tiny, interconnected crystals by nucleation agent effect shown in Figure 2 might be attributed to the up-hill diffusion of the liquid-liquid phase separation via spinodal decomposition.

**Figure 5:**
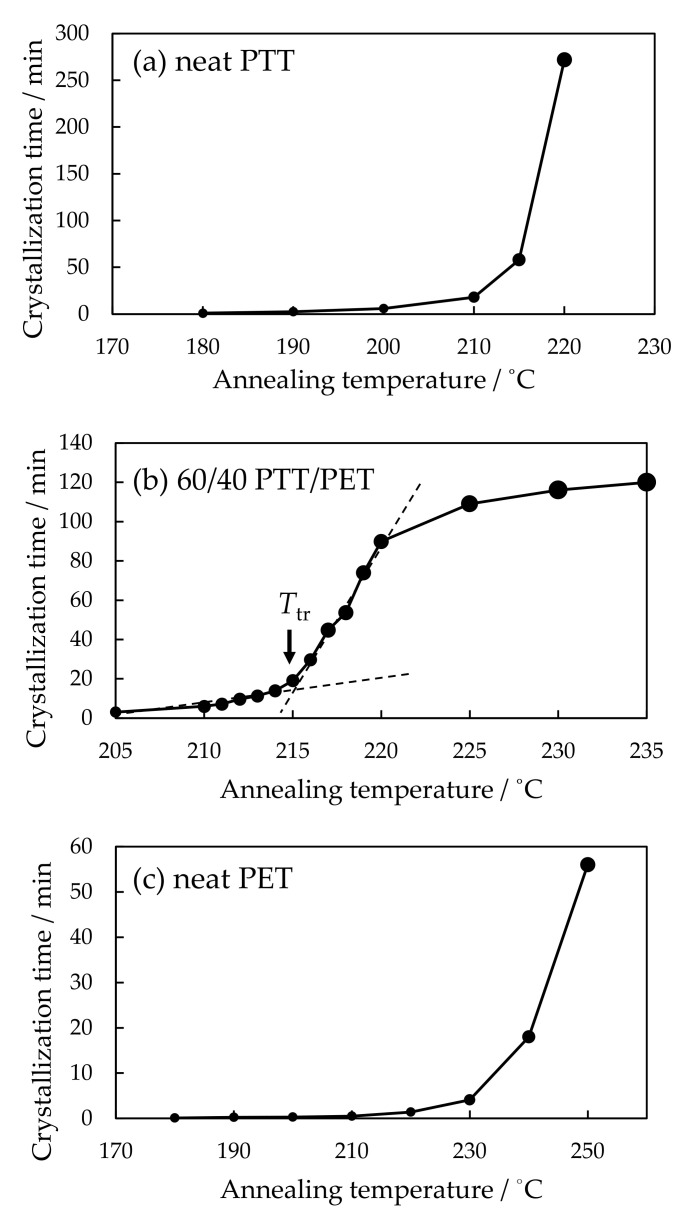
Crystallization time of (**a**) neat PTT, (**b**) 60/40 PTT/PET and (**c**) neat PET at various annealing temperatures.

In order to estimate the crystallization rate, crystallization time was obtained by subtracting the start time from the completion time of the increase of the *Q*_Hv_ in Figure 4. As the crystallization time is shorter, crystallization rate is faster. Figure 5 shows the crystallization time of the 60/40 PTT/PET and the neat component polymers at various crystallization temperatures. The crystallization time increased continuously with temperature and it increased steeply at high temperature close to the melting temperature in the neat component polymers (Figure 5a,c). On the other hand, the discontinuous gap was seen at around 215 °C in the blend, i.e., it increased steeply at around 215 °C after the gradual increase with temperature and then increased gradually (Figure 5b). This is similar to that observed in first-order transition The discontinuous gap of the crystallization time might be attributed to the phase transition at the temperature *T*_tr_= 215 °C and accelerated crystallization is suggested at the temperature below the *T*_tr_.

**Figure 6:**
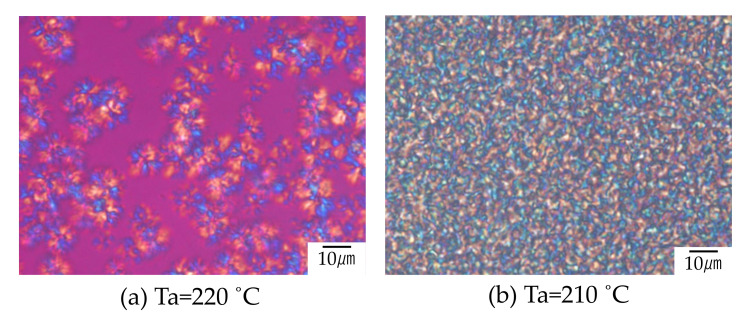
Crystalline morphology of 60/40 PTT/PET at different annealing temperatures: (**a**) Ta = 220 °C and (**b**) Ta = 210 °C.

The interesting result here is that the temperature *T*_tr_ for the discontinuous gap shown in Figure 5 corresponds to that for the change of the crystalline structure, as shown in Figure 6. Large spherulites were obtained by symmetric growth at higher temperature of 220 °C (Figure 6a). On the other hand, highly interconnected tiny spherulites were obtained at lower temperature of 210 °C (Figure 6b). Such structure change at narrow temperature region did not occur in the neat component polymers, but it was characteristic of the blends. The interconnected structure developed at lower temperature region is one of the hallmarks of liquid-liquid phase separation via spinodal decomposition, while large spherulite developed at high temperature is generally observed in the crystallization of miscible polymer blends. Thus, the results suggest existence of upper critical solution temperature (UCST) phase boundary in which miscible blend tends to phase separate at lower temperature *T*_cr_. To discuss the existence of the UCST phase boundary and the effect on the crystallization, the results of the light scattering measurements are presented in the following. 

The Vv light scattering is attributed to both the optical anisotropy and the density fluctuation. The invariant in the Vv mode *Q*_Vv_ is ascribed to both mean square optical anisotropy <*δ*^2^> and the mean-square density fluctuation <*η*^2^>. The <*η*^2^> in a neat crystalline polymer system is given by

(3) $$\;{\langle \eta \rangle }^{2}=\Phi_{S}\left(1-\Phi_{S}\right){\left(\alpha_{c}-\alpha_{a}\right)}^{2}\;$$

where $\alpha_{c}$ is the average polarizability is the average polarizability of the spherulites and *α*_a_ is the polarizability of an amorphous matrix. In a phase-separated blend of polymers A and B, the <*η*^2^> is similarly described by

(4) $$\;{\langle \eta \rangle }^{2}=\Phi_{A}\left(1-\Phi_{A}\right){\left(\alpha_{A}-\alpha_{B}\right)}^{2}\;$$

where *ϕ*_A_ is the volume fraction of A-rich phase and *α*_A_ is the polarizability of the A-rich phase [41]. Thus, Vv light scattering intensity is highest when the volume fraction of A-rich phase is 50%.

**Figure 7:**
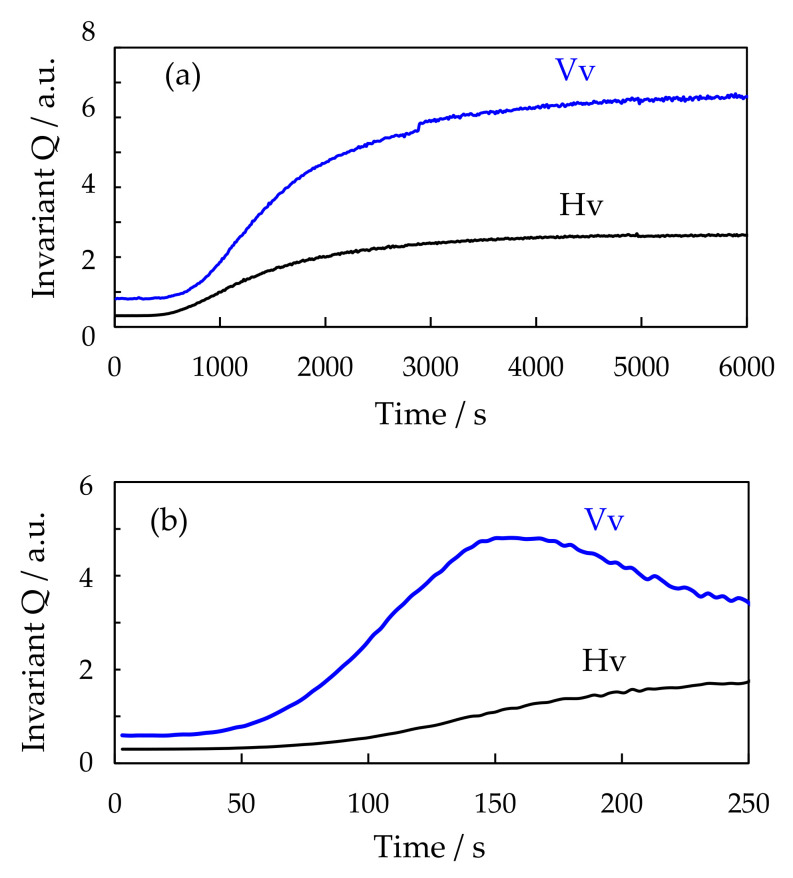
Time variation of invariant *Q*_Hv_ and *Q*_Vv_ for 60/40 PTT/PET during isothermal annealing at different temperatures: (**a**) 230 °C, (**b**) 210 °C.

Figure 7 shows the time variation of the invariants *Q*_Hv_ and *Q*_Vv_ for the 60/40 PTT/PET during isothermal annealing at 230 °C and 210 °C. Both *Q*_Hv_ and *Q*_Vv_ started to increase at the same time and gradually increased with annealing time at 230 °C (Figure 7a), indicating that the density fluctuation and optical anisotropy starts to increase at the same time. Such time variation is typical for the crystallization of polymers. In contrast, *Q*_Hv_ and *Q*_Vv_ did not start to increase at the same time, but the *Q*_Vv_ increased first and then the *Q*_Hv_ started to increase after the time lag (Figure 7b). The result suggests that liquid-liquid phase separation occurs before crystallization starts to occur due to the crystallization in the two-phase region below the UCST phase boundary.

**Figure 8:**
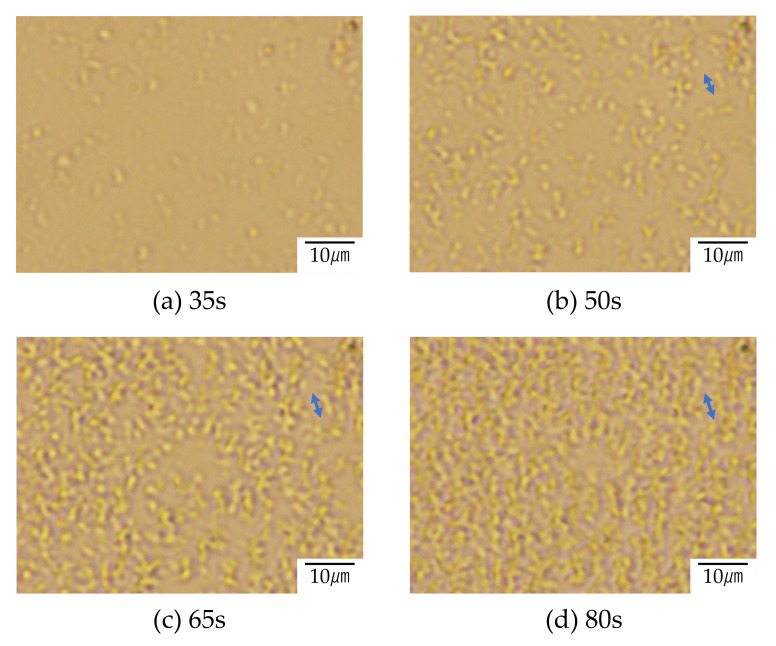
Structure development of 60/40 PTT/PET during the crystallization at 210 °C after (**a**) 35 s, (**b**) 50 s, (**c**) 65 s and (**d**) 80 s observed by optical microscope.

**Figure 9:**
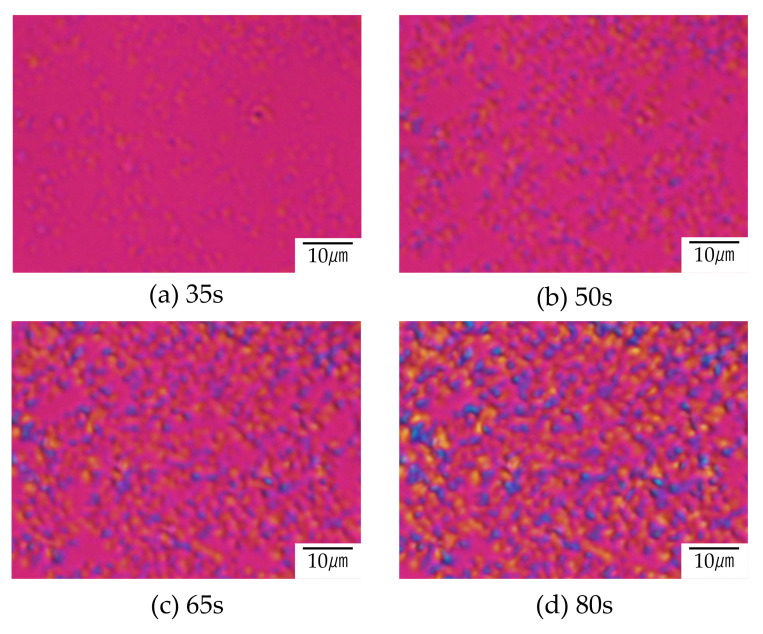
Structure development of 60/40 PTT/PET during the crystallization at 210 °C after (**a**) 35 s, (**b**) 50 s, (**c**) 65 s and (**d**) 80 s observed polarized optical microscope.

Figure 8 and Figure 9 show the structure development for the interconnected crystalline structure of the 60/40 PTT/PET observed by optical microscopy and polarized optical microscopy, respectively. Initially, spherical domains having a size of several micrometers appeared (Figure 8a), while no anisotropic structure was seen (Figure 9a). This result indicates that the spinodal decomposition precedes and the crystallization follows as suggested by light scattering measurement shown in Figure 7. The spherical domains grew asymmetrically, and their shape became distorted (Figure 8a). The distorted domains became longer over time and the neighboring domains impinged on each other along the long continuous phase (Figure 8c). The contrast of the two-phase structure became higher during the structure development. Such structure change might be attributed to the liquid-liquid phase separation via spinodal decomposition. Due to the liquid-liquid phase separation via spinodal decomposition, spherical crystal domains impinged on each other along the long continuous phase, and interconnected spherulites were obtained (Figure 9b,c). The crystallization occurs during the development of the spinodal decomposition. Owing to the up-hill diffusion by spinodal decomposition, PTT chain are forced to move from the PET-rich region to the PTT-rich region and PET chains are from the PTT-rich region to the PET-rich region. Then the nucleation of PTT and PET crystallites are induced simultaneously. This means that the crystallizable chains are supplied to the crystallites under the thermodynamic driving force for the liquid-liquid phase separation. Due to the induction of each crystallite, the crystallization rate of the blend is accelerated, as shown in Figure 4 and Figure 5.

**Figure 10:**
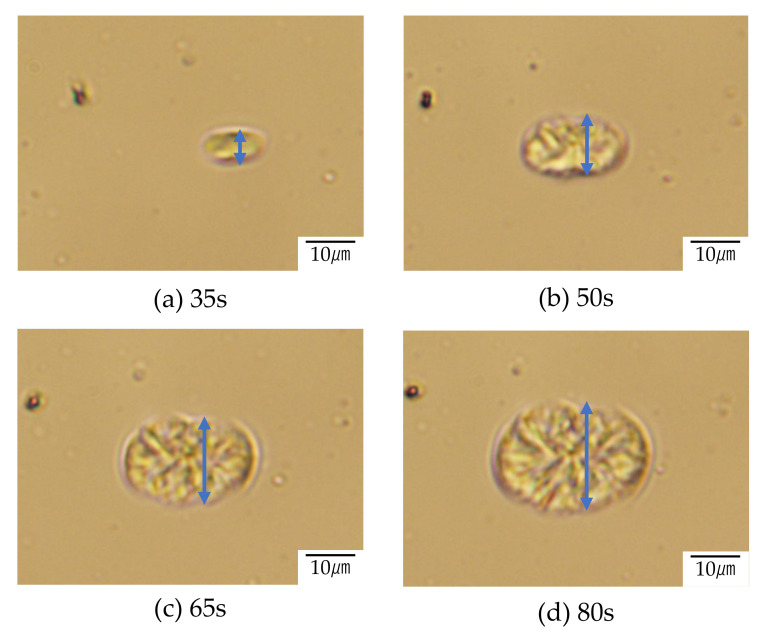
Structure development of neat PTT during the crystallization at 210 °C after (**a**) 35 s, (**b**) 50 s, (**c**) 65 s and (**d**) 80 s observed by optical microscope.

There are two causes for the accelerated crystallization in the blend, i.e., fast nucleation rate and an increase in crystal growth rate. To clarify the causes of the accelerated crystallization, the development of the crystal growth of the neat PTT in Figure 10 for comparison with that of the blend shown in Figure 8 and Figure 9. The crystal growth of the blend was faster than that of the blend; e.g., the crystal size of the blend was 3.3 μm and 5.4 μm while that of the neat PTT was 6.1 μm and 17.6 μm at 35 s and 65 s, respectively. Thus the accelerated crystallization in the PTT/PET is attributed to the enhancement of the nucleation rate as reported in the PEH/PEB blends [18,19]. The dynamic Monte Carlo simulation for liquid-liquid phase separation and crystallization suggest that crystal nucleation is enhanced at the diffuse interface between two immiscible polymers [30,31,32].

**Figure 11:**
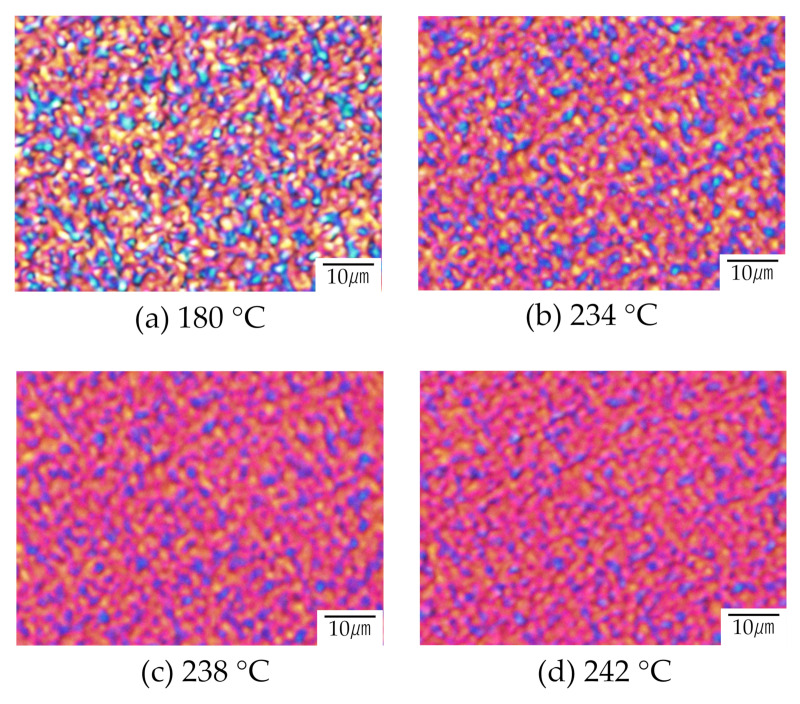
Polarized optical micrograph for the melting behavior during the heating of 60/40 PTT/PET obtained by annealing at 180 °C: (**a**) 180 °C, (**b**) 234 °C, (**c**) 238 °C and (**d**) 242 °C.

Figure 11 shows the melting behavior of the 60/40 PTT/PET with interconnected crystalline structure obtained at 180 °C. Due to the melting of the PTT crystallites during the heating, non-crystalline region colored by purplish-red became wider, but interconnected structure colored by blue and yellow remained, though higher content of PTT (60 wt%) melted at around the melting temperature of the PTT (228 °C). The result suggests that both PET and PTT phase exists due to the liquid -liquid phase separation via spinodal decomposition.

**Figure 12:**
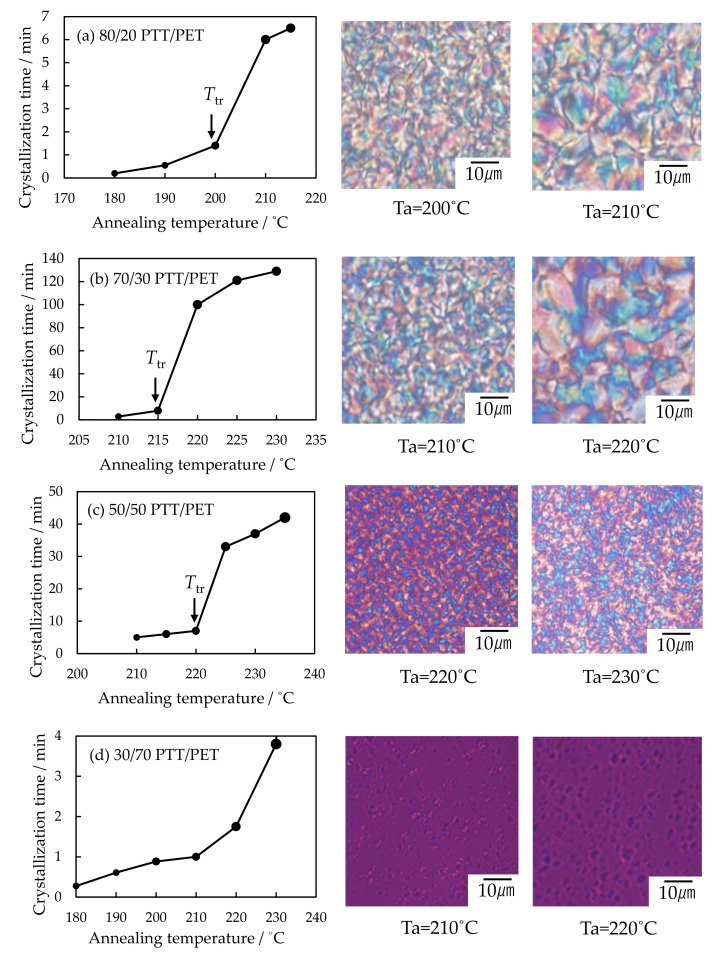
Crystallization rate at various crystallization temperatures and the crystalline morphology at around the *T*_tr_ of PTT/PET blends at various blend compositions: (**a**) 80/20, (**b**) 70/30, (**c**) 50/50 and (**d**) 30/70 PTT/PET.

As shown in Figure 12, discontinuous gap of the crystallization time was also seen at the temperature *T*_tr_ in the blend at the PET composition below 50 wt%, and the structure development was different with temperature at the boundary of the *T*_tr_. At the PET composition above 50 wt%, difference of the structure development was observed at around 210 °C in the 30/70 PTT/PET, for instance, but discontinuous gap of the crystallization time was unclear because the crystallization rate was too fast to observe the gap. 

**Figure 13:**
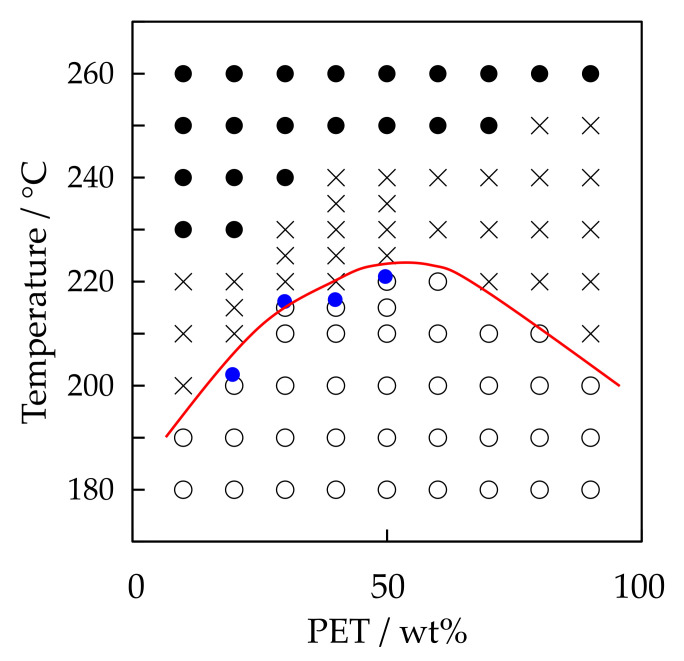
Phase diagram of the PTT/PET blends.

Figure 13 shows the phase diagram of the PTT/PET blends. As shown in Figure 6 and Figure 12, large spherulites were obtained by symmetrical growth at higher temperature region. This is indicated by the crosses. On the other hand, interconnected tiny spherulites were obtained by liquid-liquid phase separation at lower temperature region. This is indicated by the open circles. On the basis of these observations, the UCST line was drawn at the boundary for the difference of the structure development. The temperature for the discontinuous gap of the crystallization rate obtained from Figure 5 and Figure 12 was indicated by the blue filled circles. These blue circles located at around the UCST line. These results suggest that accelerated crystallization is caused by the liquid-liquid phase separation via spinodal decomposition at the temperature below the UCST phase boundary.

## 4. Conclusions

The UCST type phase boundary below the melting temperature was determined in the PTT/PET blends by the discontinuous gap of the crystallization time and the difference of the structure development. Interconnected tiny spherulites were obtained by the liquid-liquid phase separation via spinodal decomposition at the temperature below the UCST phase boundary *T*_tr_, while large spherulites were obtained by symmetric growth at the temperature above the *T*_tr_. The crystallization of the blends was accelerated due to the enhancement of the nucleation rate when the crystallization occurred at the temperature below the *T*_tr_, due to the liquid-liquid phase separation via spinodal decomposition.
